# Clinical and demographic pattern of chronic granulomatous disease (CGD) from a multicenter perspective: Malaysia’s experience over 26 years

**DOI:** 10.1186/s13223-021-00551-4

**Published:** 2021-05-17

**Authors:** Lokman Mohd Noh, Amir Hamzah Abdul Latiff, Intan Hakimah Ismail, Rahim Md Noah, Asrul Abdul Wahab, Intan Juliana Abd. Hamid, Adiratna Mat Ripen, Nasuruddin B. Abdullah, Kamarul Azhar Razali, Norzila Zainudin, Florence Bakon, Long Juan Kok, Adli Ali, Bilkis Banu SAbd Aziz, Hasniah Abdul Latif, Siti Mardhiana Mohamad, Zarina Thasneem Zainudeen, Ilie Fadzilah Hashim, Iean Hamzah Sendut, Thiyagar Nadarajaw, Faizah Mohamed Jamil, David C. E. Ng, Mohd Azri Zainal Abidin

**Affiliations:** 1 Department of Paediatrics, Hospital Tunku Azizah, Ministry of Health Malaysia, Kuala Lumpur, Malaysia; 2 Department of Paediatrics, UKM, Kuala Lumpur, Malaysia; 3 Pantai Hospital, Kuala Lumpur, Malaysia; 4 Department of Paediatrics, Faculty of Medicine and Health Sciences, Universiti Putra Malaysia, Selangor, Malaysia; 5 Universiti Kuala Lumpur, Kuala Lumpur, Malaysia; 6 Department of Medical Microbiology and Immunology, Universiti Kebangsaan Malaysia Medical Centre, Kuala Lumpur, Malaysia; 7 Primary Immunodeficiency Group, Cluster of Regenerative Medicine, Advanced Medical and Dental Institute, Universiti Sains Malaysia, Kepala Batas, Penang Malaysia; 8 Primary Immunodeficiency Unit, Institute for Medical Research, Kuala Lumpur, Malaysia; 9 Formerly At International Islamic University, Kuantan, Malaysia; 10 Institute for Medical Research, Kuala Lumpur, Malaysia; 11 Al Islam Specialist Hospital, Previously At Institute of Pediatrics, Hospital Kuala Lumpur, Kuala Lumpur, Malaysia; 12 Sunway Medical Centre, Petaling Jaya, Selangor Malaysia; 13 Institute of Pediatrics, Hospital Kuala Lumpur, Kuala Lumpur, Malaysia; 14 KPJ Kuching Specialist Hospital, Kuching, Sarawak Malaysia; 15 Sarawak General Hospital, Kuching, Sarawak Malaysia; 16 Department of Paediatrics, Faculty of Medicine, Universiti Kebangsaan Malaysia Medical Centre, Kuala Lumpur, Malaysia; 17 Cluster of Life Sciences, Advanced Medical and Dental Institute, Universiti Sains Malaysia, Kepala Batas, Penang Malaysia; 18 Gleneagles Hospital, Kuala Lumpur, Malaysia; 19 Faculty of Medicine, University Malaya, Kuala Lumpur, Malaysia; 20 Hospital Sultanah Bahiyah, Alor Setar, Kedah Malaysia; 21 Hospital Serdang, Serdang, Selangor Malaysia; 22 Hospital Tuanku Ja’afar, Seremban, Negeri Sembilan Malaysia; 23 Sarawak General Hospital, Kuching, Sarawak Malaysia

**Keywords:** Chronic granulomatous disease, Malaysia, Clinical, Demographic pattern, *Chromobacterium violaceum*, CD4 + lymphopenia

## Abstract

**Background:**

A retrospective review of clinical manifestations and demographic pattern of patients diagnosed as chronic granulomatous disease (CGD) from 7 hospitals in Malaysia. An analysis of the available database would establish clinical characteristics, diagnoses and outcome including microbiologic pattern. Studying the demography allows us to document the occurrence of CGD amongst multiethnic groups and its geographical distribution for Malaysia.

**Methods:**

Data from the Malaysia Primary Immunodeficiency Network (MyPIN) with cases of CGD diagnosed from 1991 until 2016 were collated and analysed.

**Results:**

Twenty patients were diagnosed as CGD. Males (N = 13, 65%) outnumber females (N = 7, 35%). CGD is commonest amongst the Malays (65%) followed by the Chinese (15.0%), Indians (10.0%) and natives of Borneo (10.0%), reflecting the ethnic composition of the country. The mean age of diagnosis was 3.7 years. There was a positive family history in 40% of the cases. Abscess was the main presenting feature in 16 patients (80%) with one involving the brain. Pneumonia occurred in 10 (50%) and one with complicated bronchiectasis. Catalase-positive bacteria were the most commonly isolated pathogen with *Chromobacterium violaceum* predominating (N = 5, 25%) with consequent high mortality (N = 4, 80%). All CGD patients with *C. violaceum* infection displayed CD4 + (T helper cells) lymphopenia.

**Conclusion:**

This study has shown CGD occurs in the major ethnic groups of Malaysia. To the best of our knowledge, this is the first and the largest series of chronic granulomatous disease in South East Asia which may be reflective of similar clinical pattern in the region. *C. violaceum* infection is associated with a higher mortality in CGD patients in Malaysia. All the CGD patients with *C. violaceum* infection in this patient series displayed CD4 + (T helper) lymphopenia. We recorded rare clinical manifestation of CGD viz. brain abscess and bronchiectasis.

## Background

Chronic granulomatous disease (CGD) is a genetic disorder resulting from the defect of one of the subunits of enzyme NADPH oxidase. It is responsible for the production of the reactive oxygen species (ROS) by the phagocytes to kill the engulfed pathogen. Defects of this enzyme predispose the susceptible individual to infection mainly by catalase-positive organisms. The mode of inheritance may be further classified into X-linked inheritance (mutation in CYBB gene) and autosomal recessive inheritance (mutation in either CYBA, CYBC1, NCF1, NCF2 and NCF4 genes) [1].

The prevalence of the disease is not well described in many developing countries. A study in the United States (US) estimated the incidence of CGD is between 1 in 200,000 to 1 in 250,000 live births [2]. A systematic review on published primary immunodeficiency disease (PID) in Malaysia showed that CGD was the third most common reported form of PID, which is comparable with Singapore’s PID cohort [3]. Chronic granulomatous disease cases in Malaysia were first reported in 1994 diagnosed in two unrelated boys [4] based on nitroblue tetrazolium dye test (NBT) and chemiluminescence assay (CA); and this would have been the first report from South East Asia (SE Asia). Few countries in SE Asia (Malaysia, Singapore and Thailand) have published reports of case series of PID which included CGD [3–7]. This report would be the first patient series in SE Asia, revealing some inherent differences in the clinical and demographic patterns from the Europe, America and Asia [2, 8–10, 18]

## Methods

Malaysia Primary Immunodeficiency Network (MyPIN) is a collaborative network of clinical immunologists, immunopathologists, scientists and general pediatrician/physicians managing primary immunodeficiency patients in both government and academic institutions. Patients referred for recurrent infections with a diagnosis of primary immunodeficiency seen in seven hospitals in Malaysia between 1986 and 2016, were entered into data sheets by the corresponding author from the beginning. The seven hospitals were: Institute of Pediatrics, Hospital Kuala Lumpur, Hospital Pantai Kuala Lumpur, Hospital Serdang Selangor, Sarawak General Hospital Kuching, Hospital Canselor Tunku Muhriz, UKM Cheras, University of Malaya Medical Centre, Kuala Lumpur, Hospital Sultanah Bahiyah, Alor Setar Kedah. In the absence of a National centralized registry for the country this is the most optimal system of record to ensure PID data is preserved. All clinical and immunological data of patients with diagnosis of chronic granulomatous disease were retrieved and analyzed. The diagnosis of CGD was based on clinical features and abnormality of phagocytic function, inability to generate reactive oxygen species [10]. Only 20 could be retrieved with phagocytic function assay that included either NBT and/or CA and/or dihydrorhodamine 123 (DHR) assay in the participating immunology laboratories. NBT and/or CA were the only assays that could be done up to 2010; subsequent diagnostic tests including DHR assay were done by the Advanced Medical and Dental Institute (AMDI), Universiti Sains Malaysia, Penang (AMDI first started this test in 2011 [11]), Institute for Medical Research, Kuala Lumpur and Universiti Putra Malaysia, Serdang. Four patients had mutational assays done (NCF1 and CYBB). Both NBT and CA were done in 4, chemiluminescence assay in 19 and DHR 123 in 4 patients.

### Dihydrorhodamine (DHR) test

Heparinized whole blood was incubated with the various stimuli (unlabeled opsonized E. coli, PMA and fMLP) at 37 °C. A sample without stimulus served as negative background control. Formation of the reactive oxidants during the oxidative burst was monitored by the addition and oxidation of DHR123. The reaction was stopped by addition of lysing solution, and after one washing step with wash solution, DNA staining solution was added to exclude aggregation artifacts of bacteria or cell.

### Flow cytometry

DHR samples were then analyzed by using BD FACSCanto™ II. Ten thousand viable granulocytes determined by forward and side scatterplot were collected, and FL2 (585 nm) was analyzed. A sample from healthy subject was used as a positive control for each assay. The percentages of cells having produced reactive oxygen radicals were then analyzed as well as the stimulation index (SI) was calculated as the ratio of geometric mean channel fluorescence intensity of PMA-stimulated and unstimulated granulocytes.

### Genetic test

Genetic tests were not universally available for patients in Malaysia. Only 4 were performed and were mainly performed as a research activity, with 3 of the earlier samples for analysis sent to centers overseas.

## Results

### Demography of patients

Twenty patients were diagnosed with CGD either by functional assays of the phagocyte function that included 13 males and 7 females*.* Genetic mutational assays were available for four patients (NCF1 = 2, CYBB = 2). However, defect in p47phox protein expression was documented in 6 patients, which were suggestive of autosomal recessive CGD (NCF1 gene mutation).

The mean age onset of symptoms was 2.16 ± 3.13) years old. The patients were diagnosed between the age of 0.3 to 12 years*,* with a mean age of diagnosis of 3.7 ± 3.95) years old. Ethnic distribution showed Malays (N = 13, 65%) was the predominant race, followed by Chinese 15% (N = 3), Indians 10% (N = 2) and Natives of Borneo 10% (N = 2). It is remarkable that 5 (25%) of the CGD patients were from different families residing in Sarawak; a state in East Malaysia with a population that constitute only 9% of total Malaysia’s population. Family history of early death for possible of PID in the first degree relative was obtained in 8 patients (40%). Consanguinity was noted in two patients (10%). Table 1 represents the clinical and demographic presentations of the patients with CGD in Malaysia.

**Table 1:** Clnical and demographic characteristics of CGD patients in Malaysia 1991–2016

Patient	Gender	Ethnic	Age(y) onset.	Age (years) diagn.	E Sib death	Clinical features	0rganism	0utcome	Neutrophi defect	DHR abn	Gene mutatiojn	Protein abn
P01	F	M	3	7	Y	Abscess skin, eczema, septicemia, retinitis pigmentosa	N menigitides, *C violaceum*	Alive	CA NBT	nd	NCF1	p47 phox
P02	M	M	na	1.3	Y	Abscess skin, cellulitis, pneumonia, lymphadenitis, septicemia, cholecystitis	*C violaceum*	*Died*	CA NBT	nd	nd	nd
P03	M	C	11	11	Y	pneumonia	norcardia	Alive	CA	nd	nd	p47 phox
P04	F	C	na	5	Y	Recurrent. lymphadenitis( cervical )	nil	Loss FU	CA	nd	nd	p47 phox
P05	M	M	na	0.4	Y	Abscess (skin, Liver), jaundice	*Staph*, E Coli	*Died*	CA NBT	nd	nd	nd
P06	M	M	na	0.9	Y	Abscess skin, pneumonia, septicemia, hepatomegaly,splenomegaly	*C violaceum*	*Died*	CA	nd	nd	nd
P07	F	M	na	0.6	n	A bnormal facies, abscess skin, hepatomegaly,splenomegaly	nil	Loss FU	CA	nd	nd	nd
P08	M	I	3.7	5	n	Abscess skin	na	Alive	CA	nd	nd	nd
P09	F	M	0.1	0.3	n	Abscess scalp, oral thrush,	candida	Loss FU	CA	nd	nd	nd
P10	F	M	na	0.9	na	Abscess skin gluteal region	na	Loss FU	CA	nd	nd	nd
P11	F	M	0.3	1	na	Pneumonia	na	Loss FU	CA	nd	nd	nd
P12	M	NB	0.3	2	na	Abscess( (iver,spleen), pneumonia	B peudomallei (titres)	Alive HSCT	CA	nd	nd	p47 phox
P13	M	I	3	12	n	Abscess skin submental, inguianal, recurrent eye infections	na	loss FU	nd	+	nd	p47 phox
P14	M	M	5	5	na	Abscess brain, gangrenous toe, hepatomegaly,splenomegaly	*C violaceum*	*Died*	CA	nd	NCF1	p47 phox
P15	F	M	0.6	4.5	n	Abscess (periosteum, spleen), arthritis	na	Loss FU	CA	nd	nd	nd
P16	M	NB	0.67	1.9	n	Abscess spleen,pneumonia, hepatomegaly,splenomegaly	RSV, mycoplasma	Alive	CA	nd	nd	nd
P17	M	M	0.08	1.6	Y	Abscess speen, pneumonia, disseminated TB	nil	Alive	CA	+	CYBB	nd
P18	M	C	0.25	0.9	n	Lymphadentis TB, pneumonia, meningitis, meliodosis hepatomegaly splenomegaly	*Klebsiella* sp. *Staph areus*, B pseudomallei(titre)	Alive	CA	+	CYBB	nd
P19	M	M	na	12	Y	Absccess skin, bronchiectasis	na	Loss FU	CA	+	nd	nd
P20	M	M	0.08	0.6	n	Abscess skin, pneumonia, osteomyelitis, hepatomegaly,splenomegaly	*Kleibsella*, *C violaceum, * *E coli.*	*Died.*	CA	+	nd	nd

E sib death (early sibling death), yes(y), no(n), na (not available)

CA Chemiluminesc. Assay, + DHR defect present, nd (not done)

Ethnic groups: M (Malay) C (Chinese), I (Indian), NB (North Borneo indigenous group); Age in Years ; Outcome:Loss FU(loss to follow up)

## Clinical manifestations and types of infections

The most common clinical presentation of the patients was abscesses, (N = 16, 80%; skin = 10, spleen = 3, liver = 3, bone = 1, brain = 1, muscle = 1) followed by pneumonia (N = 10, 50%) with one complicating as bronchiectasis. Hepatosplenomegaly (30%) and lymphadenitis (15%) were the next common findings. Other sites of infections were listed in Table 2. *Mycobacterium tuberculosis* infection was diagnosed based on clinical grounds.

**Table 2:** Infections manifestation of CGD patients from Malaysia

Site of Infections	Number of patients (%)
Abscess	16 (80)
Skin	10
Spleen	3
Liver	3
Bone	1
Brain	1
Muscle	1
Lung infections (pneumonia/abscess)	10 (50)
Hepatosplenomegaly	6 (30)
Septicemia	3 (15)
Meningitis	1 (5)
Lymphadenitis	3 (15)
Eye infection	1 (5)
Retinitis pigmentosa & discoid lupus	1 (5)
Osteomyelitis	1 (5)
Arthritis	1 (5)
Oral candidiasis	2 (5)

### Microbiological and immunological profiles

The microbiological profile of the patients was predominantly of the catalase-positive bacteria as shown in Table 3. *Chromobacterium violaceum* were isolated from 5 (25%) patients. Melioidosis was diagnosed in 2 children based on high titers to *Burkholderia pseudomallei*. *Staphylococcus spp.* was isolated in 2 patients (one with *Staphylococcus aureus* and one with *Staphylococcus epidermidis*) from skin abscesses specimens. Bacterial infections (N = 13) predominated over fungal infections (N = 3).

**Table 3:** Types of microorganism in CGD patients from Malaysia

Organism	Frequency n (%)
*Chromobacterium violaceum*	5 (25)
*Burkholderia pseudomallei *(titre)	2 (10)
*Staphylococcus spp.*	2 (10)
*Klebsiella*	2 (10)
Candida*	2 (10)
Nocardia	1 (5)
RSV	1 (5)
*Neisseria meningitidis*	1 (5)
*E coli*	1 (5)
*Mycoplasma*	1 (5)

*Oral candidiasis in both patient

The immunological parameters of the patients are illustrated in Table 4. Serum immunoglobulin showed hypergammaglobulinemia with elevated IgG, IgA and IgM in 73, 86 and 53% of the cases, respectively. Elevated lymphocyte subsets were also observed, 46% of the CGD patients had elevated CD3 + lymphocyte counts, 27% with high CD4 + and 36% with high CD8 + lymphocyte counts. B cells were elevated in only 20% of the cases. CD4 + lymphopenia was seen in 36.4% of the cases, with low CD3 + and CD8 + lymphocyte counts observed in 6.7% and 18.2% of CGD patients, respectively. Low immunoglobulin levels were not much affected, ranges between 0 and 6% of the cases only. Interestingly, all the 4 (100%) CGD patients with CD4 + lymphopenia had been infected with *C. violaceum.*

**Table 4:** Proportion(%) of CGD with high or low Immune parameters in Malaysian patients with CGD

Immune parameters	High levels*	Normal levels	Low levels**
	N (%)	N (%)	N (%)
Lymphocyte subsets			
CD3+/T cells (N = 15)	7 (46.6%)	7 (46.6)	1 (6.7%)
CD4+/Th cells (N = 11)	3 (27.3%	4 (36.4)	4. (36.4%)
CD8 /Tc cells (N = 11)	4 (36.4%)	5 (45.5)	2 (18.2)
B cell (N = 15)	3 (20 %)	8 (53.3 %)	4 (26.6% )
Serum immunoglobulins			
IgG (N = 15	11 (73)	3 (20)	1 (6)
IgA (N = 14)	12 (86)	2 (14.2)	0
IgM (N = 15)	8 (53)	7 (47)	0

*Above normal range for age matched healthy children

**Below normal range for age matched healthy children

Normal & high level IgG. 14/15 (93.3%) CD3+ cells 14/15(93.3%)

IgA. 14/14. (100%) CD4+ cells 7/11 (63.6%)

IgM. 15/15. (100%) CD8+ cells 9/11 ( 81.8%)

### Management and outcome of the patients

Of the patients that could be followed up, 5 (25%) died, with 4 patients displaying *C. violaceum* infection. It appeared that *C. violaceum in CGD* was associated with high mortality rate (80%)*.* Most of the patients received trimethoprim-sulfamethoxazole and itraconazole as an antimicrobial of choice for prophylaxis. None received interferon (IFN) gamma therapy as it was not readily available locally. One patient (P12) received allogeneic hematopoietic stem cell transplantation (HSCT) and is still alive 12 years later [13].

## Discussion

The earliest published report of CGD in Malaysia as case reports was in 1994 compared to India in 1999 and Taiwan in 2000 [14, 15]. There was another report of CGD in Malaysia in 2012 [16]. Herein, we report 20 cases of CGD from 7 hospitals located both in Peninsular and East Malaysia. As with most South East Asian countries, facilities, and resources for PID care in Malaysia are limited; mutational analysis was not available locally until the last decade of which samples had to be sent overseas.

To the best of our knowledge this report of CGD patient series is the first and the largest from SE Asia. The other large series from Thailand is actually a PID series of 67 patients which included 6 CGD patients [7]. The mean age of diagnosis for our patients was 3.7 years, much later than Sri Lanka (1.6 years), and China (2.24 years), but earlier than India (4.6 years) [8, 9, 17].

Chronic granulomatous disease occurs more commonly in males and with X-linked mode of inheritance as seen in two large studies from Europe and the US, where males predominate with the former at 81% (351 out of 429) and the latter at 70.5% (259 out of 368 patients), respectively [2, 19]*.* Similar occurrence was seen in France (88 out of 97 patients, 90.7%) [18]. Predominant mode of autosomal recessive inheritance is seen in reports from India, Iran, Turkey, Israel and Saudi Arabia [9, 21–24]. It is noteworthy that a study done in a province in Saudi Arabia reported that all of their patients were autosomal recessive inheritance with a point prevalence of 6.4 per 100,000, the highest being reported up to 2009 in the literature [24]. Positive family history is seen in 40% of the Malaysian series which is higher in large reports from China 28% or Italy 35% [17, 25]. We could not conclude that there is a preponderance for autosomal recessive CGD (AR CGD) over X-linked CGD (XL CGD) in our cohort in view of limited genetic mutational analysis performed. Interestingly, 4 of 6 (66%) cases were with p47phox mutation, a marker of AR CGD defect, were males. Further data on mutational analysis will be needed to confirm if indeed there is a preponderance of AR CGD over XL CGD in Malaysia.

Abscesses are the most common presentation, unlike most other series where pneumonia is the commonest [2, 18, 20, 22]. Pneumonia constitutes only 50% in our series making it the second most common presentation. One of the CGD patients had bronchiectasis as a sequela. Bronchiectasis is not a common finding amongst those with lung infections [21]. Brain abscess, a very uncommon presentation, is present in our series. Such presentation has been reported in another series [2, 23]. Lymphadenitis was the most common clinical presentation in some CGD series. However, in the Malaysian cohort, it is the third most common presentation [8, 19, 21, 24]. Hepatosplenomegaly is also one of the common physical findings (30%) in this series.

In term of infectious organisms, patients with CGD are prone to catalase-positive bacteria including *C. violaceum.* It is a gram-negative facultative anaerobe which is catalase positive dominating a variety of ecosystem in tropics and sub-tropical regions. It is a rare infection in human with less than 200 cases reported in the literature [27, 28]. However, they can have a fulminating presentation with a high mortality [29]. Infection in human was first documented in Malaysia (1927), later Singapore, Vietnam, Thailand, Sri Lanka, Taiwan, Hong Kong, India, Australia, Southeast region of the US and Latin America (Brazil /Argentina) [28, 31].

Earlier reports indicated that *C. violaceum* infection in CGD patients as being rare with only one of 368 patients between 1993 and 1997 reported in the US Registry [2]. It is notable that Malaysia had 2 case reports of *C. violaceum* in CGD patients, first in 1994 and second in 2008 [4, 26]. More cases were reported in a review of children with invasive *C. violaceum* infection between 1971 and 2005 by Sirinavin et al., whereby out of 25 children with invasive *C, violaceum* infection, 36% were afflicted with CGD [30]. However, when considering proportion of CGD patients with *C. violaceum* infection, it was estimated at 42.9% for Thailand [7] and and 25% for Malaysia [present study]. In the present study, CGD patients are likely to succumb to *C. violaceum* infection. Mortality for all *C. violaceum* infected patients varies from 53 to 80%, with higher risk in disseminated form of infection [29]. The mortality rate of CGD patients with *C. violaceum* infection is very much lower (7%) in the Southeast US [27, 31] as compared to Malaysia at 80% (present study).

The next common infection was *Burkholderia pseudomallei*, a Gram-negative bacillus commonly found in soil and muddy water, presenting as melioidosis. Both of our patients with this infection survived with one having undergone HSCT. Other studies reported *Staphylococcus spp.* as the most common organism isolated among their CGD patients [18, 19]. However, *Staphylococcus spp.* infection only constitutes 10% of our series. *Salmonella spp.* was noted as the most common pathogen isolated from CGD patients with septicemia [2], however, this is not seen in our cohort.

Several studies have indicated fungal infections as the predominant organisms in their CGD patients with *Aspergillu*s spp. being the predominant fungi isolated [9, 22, 23]. However, *Aspergillus* spp. was not isolated in our patients’ samples. Instead, *Candida spp.* and *Nocardia* were isolated in our laboratory*.*

*Mycobacterium tuberculosis* infection afflicted a significant proportion of CGD in Sri Lanka, China and South East Asia [8, 17, 23]. Pulmonary tuberculosis is seen in 77% of CGD in Sri Lanka and 31.7% of CGD in Iran [8, 21]. Tuberculosis did not occur as frequently in our series with only 2 (10%) recorded, each of them had lymphadenitis and the other had a disseminated form of tuberculosis. Complications of BCG vaccination are also encountered in CGD case series as in 2 reports, with 53 and 64% of CGD vaccinated respectively resulting with infectious complication mainly localized around the injection sites [17, 32].

Patients with long standing CGD may develop non-infectious inflammatory complications. Previous studies had indicated various non-infectious inflammatory complications that occurred at different sites of the body. The manifestations included autoimmune manifestations, organ obstruction and granuloma formation. In one study, autoimmune phenomena occurred in about 50% of their CGD patients and the manifestations included inflammatory bowel disease, reactive arthritis, idiopathic thrombocytopenia and autoimmune hepatitis [22]. Lupus syndrome was also reported in some of the patients [2]. We recorded one patient with retinitis pigmentosa and discoid lupus. Chorioretinitis had been reported in the case series from Israel [23]. The non-infectious inflammatory complications should be actively sought out during the serial clinic follow ups.

The serum immunoglobulin level is typically hypergammaglobulinemic in CGD which is ascribed to infections by bacteria and fungi [33, 34]

An interesting facet of our lymphocyte subset analysis revealed that all of the 4 (38%) of CGD with CD4 lymphopenia ended in a demise with *C. violaceum* infection. We cannot hypothesize whether *C. violaceum* is the cause of or the effect of CD4 + lymphopenia in this Malaysian CGD series. The answer would come from further studies.

Prevention of infectious complications of CGD includes prophylaxis with appropriate antimicrobial agents; prophylaxis which includes trimethoprim-sulfamethoxazole at 5 mg/kg/day (based on TMP component) and itraconazole at 5 mg/kg/day up to 100 mg daily (if body weight < 50 kg), or 200 mg daily if body weight > 50 kg [36]. Interferon gamma is not available in Malaysia. The frequency of allogeneic HSCT as curative treatment option for CGD has increased since 2006 worldwide. The largest retrospective review on the outcome of HSCT on 712 patients with CGD demonstrated good survival outcome, low incidence of graft failure and mortality in all ages [37].

The allogenic HSCT in Malaysian government healthcare service has just recently started for PID in Malaysia and a patient (P12) from this cohort was successfully underwent HSCT with a matched sibling donor [13].

The overall survival of CGD is 90% stretched well to adulthood [25]. Others quoted as 81% with long term survival [18]. However, the survival rate is lower in developing countries. The mortality of the patients that we could follow up was 25% which was lower than India (35%) and Sri Lanka 38% [ but higher than the European cohort 20%] [8, 9, 18]. Previous study demonstrated strong association between residual related reactive oxygen intermediates (ROI) and survival of patients with CGD, where those with little residual production of ROI may fare worse in the severity of illness and survival outcome [38]

## Conclusion

We have shown that CGD is prevalent in Malaysia and that similar prevalence could occur in South East Asia region. Recurrent abscesses are more frequently observed than pneumonia in Malaysia. *Chromobacterium violaceum* infection and melioidosis are being seen more often in tropical region with heavy rainfall throughout the year. A high index of suspicion is needed to detect *C. violaceum* infection in a CGD child who is admitted in severely ill state. Conversely, diagnosis CGD should be excluded when confronted with a previously healthy child with *C. violaceum* sepsis in SE Asia region. Determining lymphocyte immunophenotype for idiopathic CD4 + lymphopenia in CGD with *C. violaceum* infection has added value for its association with mortality.

## Data Availability

All data generated or analyzed in this study and included in this article are available from the corresponding author on a reasonable request.
